# Mechanical analogue for cities

**DOI:** 10.1098/rsos.220943

**Published:** 2023-03-08

**Authors:** Nicos Makris, Gholamreza Moghimi, Eric Godat, Tue Vu

**Affiliations:** ^1^ Department of Civil and Environmental Engineering, OIT, Southern Methodist University, Dallas, TX 75276, USA; ^2^ Data Science and Research Services, OIT, Southern Methodist University, Dallas, TX 75276, USA

**Keywords:** urban resilience, human mobility, Brownian motion, ensemble averages, creep compliance, statistical mechanics

## Abstract

Motivated from the increasing need to develop a science-based, predictive understanding of the dynamics and response of cities when subjected to natural hazards, in this paper, we apply concepts from statistical mechanics and microrheology to develop mechanical analogues for cities with predictive capabilities. We envision a city to be a matrix where cell-phone users are driven by the city’s economy and other incentives while using the collection of its infrastructure networks in a similar way that thermally driven Brownian particles are moving within a complex viscoelastic material. Mean-square displacements of thousands of cell-phone users are computed from GPS location data to establish the creep compliance and the resulting impulse response function of a city. The derivation of these time-response functions allows the synthesis of simple mechanical analogues that model satisfactorily the city’s behaviour under normal conditions. Our study concentrates on predicting the response of cities to acute shocks (natural hazards) that are approximated with a rectangular pulse; and we show that the derived solid-like mechanical networks predict that cities revert immediately to their pre-event response suggesting an inherent resilience. Our findings are in remarkable good agreement with the recorded response of the Dallas metroplex following the February 2021 North American winter storm.

## 1. Introduction

Cities are complex systems that traditionally have been generating creativity, growth, leadership and power together with economic, intellectual and social wealth. They are complex networks or even networks of complex networks [1–3] including the transportation infrastructure (roads, bus-lines and a subway network when available), water utility networks, the electricity grid, telecommunication networks (telephone and Internet) together with overriding social networks [4–6]. All these complex networks have been created and upgraded independently during different times with no apparent common design principles. Urbanization is an ever-growing, pressing challenge. As recently as 1950, only 30% of the world’s population lived in cities. Today more than 50% is urbanized, and by 2050 more than 3/4 of the world’s population is expected to live in cities [7,8]. As cities continue to grow, many of them along the coasts of continents which are prone to natural hazards, urban resilience has become a subject of interest in various disciplines [9–13]. The many challenges associated with rapid urban growth, community resilience and sustainable development are receiving increasing attention; yet, in practice, they have been treated as independent issues [14]. Linear *per capita* indicators that conflate distinct dynamic features pertinent to the dynamics specific to each city are typically used to assess these growing challenges; however, it appears these fail to yield direct measures of the impact that hazards can have on cities (such as earthquakes or hurricanes) in association with the need to quantify urban resilience and functional recovery [9,15].

Several parallels have been suggested between cities and other complex systems—from biological organisms [16–19] to insect colonies [20] and ecosystems [21]. Cities, while they appear as web-like structures, are much more than biological organisms or anthills, as they involve perpetual exchanges among their citizens who in their majority are productive, creative and innovative individuals, striving for upward mobility and social interaction. Accordingly, while the functionality of cities is served with the superposition of a variety of large-scale, complex networks (transportation, water, electricity, telecommunication) with no apparent common design principles, it appears that cities display a difficult-to-interpret self-organizing behaviour that leads to deterministic patterns that can be possibly described with an emerging mechanism yet to be identified [4,8,22]. Past studies [23–25] have uncovered that, in contrast to the random trajectories predicted by the initially proposed random walk models [26], human trajectories show a high degree of temporal and spatial regularity.

Part of the success of natural sciences hinges upon reductionism where analysis frameworks and methodologies are developed for predicting the emergent macroscopic behaviour of a system by monitoring and analysing the behaviour and/or properties of its constituents. For instance, in the emerging field of microrheology, the bulk frequency- and time-response functions of complex viscoelastic materials are inferred by monitoring the thermally driven Brownian motion of probe microspheres immersed within the viscoelastic material and subjected to the perpetual random forces from the collision of the molecules of the viscoelastic material [27–37].

Building on past studies on human mobility patterns [23–25] which uncovered that citizens exhibit characteristic travel distances in association with a significant probability to return to a few highly frequent locations—a behaviour that is reminiscent to particles in a potential well; in this work, we envision a city to be a matrix where people (cell-phone users) are driven by the city’s economy and other personal and societal driving incentives while using the collection of its infrastructure networks in a similar way that thermally driven Brownian probe particles are moving within a complex viscoelastic material [27–37]. Mobile phones carried by the citizens of an urban centre offer rich data on human mobility patterns and we show that the ensemble averages of human GPS location data reveal information that characterizes the dynamics of the entire urban centre. Upon computing the mean-square displacements (MSD–ensemble averages) of the recorded time histories of the paths of a large number of citizens of a given city, we propose deterministic mechanical networks with a creep compliance that is proportional to the measured MSD. The response analysis of the proposed solid-like networks offers new insights on the mechanics of urban centres and their emergent inherent resilience within the context of *engineering resilience* [38].

Meerow *et al.* [13] identify several variations of the definition of urban resilience in the engineering, business/finance and social science literature. Our study concentrates on a *single-state equilibrium* (or a steady-state regime at equilibrium) which refers to the capacity of a system to revert to its post-disturbance equilibrium state [38]; therefore, adopts the generic definition of engineering resilience—that is *the capacity of a system to recover its initial state and resume normal activity after a shock* [39–41] and hinges upon the premise that a dependable indicator of the engineering resilience of a city [38] is whether the average mobility pattern of its citizens following an acute shock matches the average mobility pattern before the shock. Our work contributes to a quantitative, science-based, predictive understanding of the dynamics and response of cities to acute shocks [4,8] and concludes that large cities of average population density following an acute shock revert immediately to their initial steady-state, pre-event behaviour.

## 2. Ensemble averages of GPS locations of cell-phone users (IDs)

**Figure 1.**
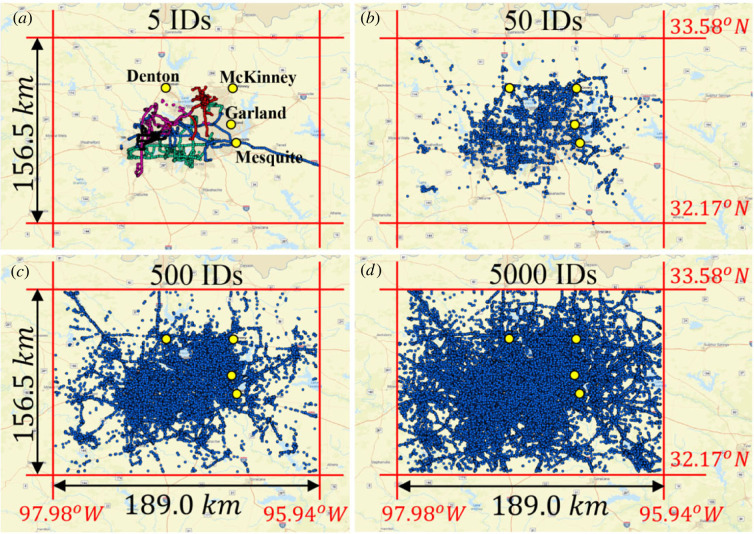
GPS locations of anonymous cell-phone users (IDs) in the greater Dallas metroplex recorded during February and March 2021: (*a*) 5 IDs; (*b*) 50 IDs; (*c*) 500 IDs; (*d*) 5000 IDs; The yellow dots are nearby cities mentioned in (*a*).

**Figure 2.**
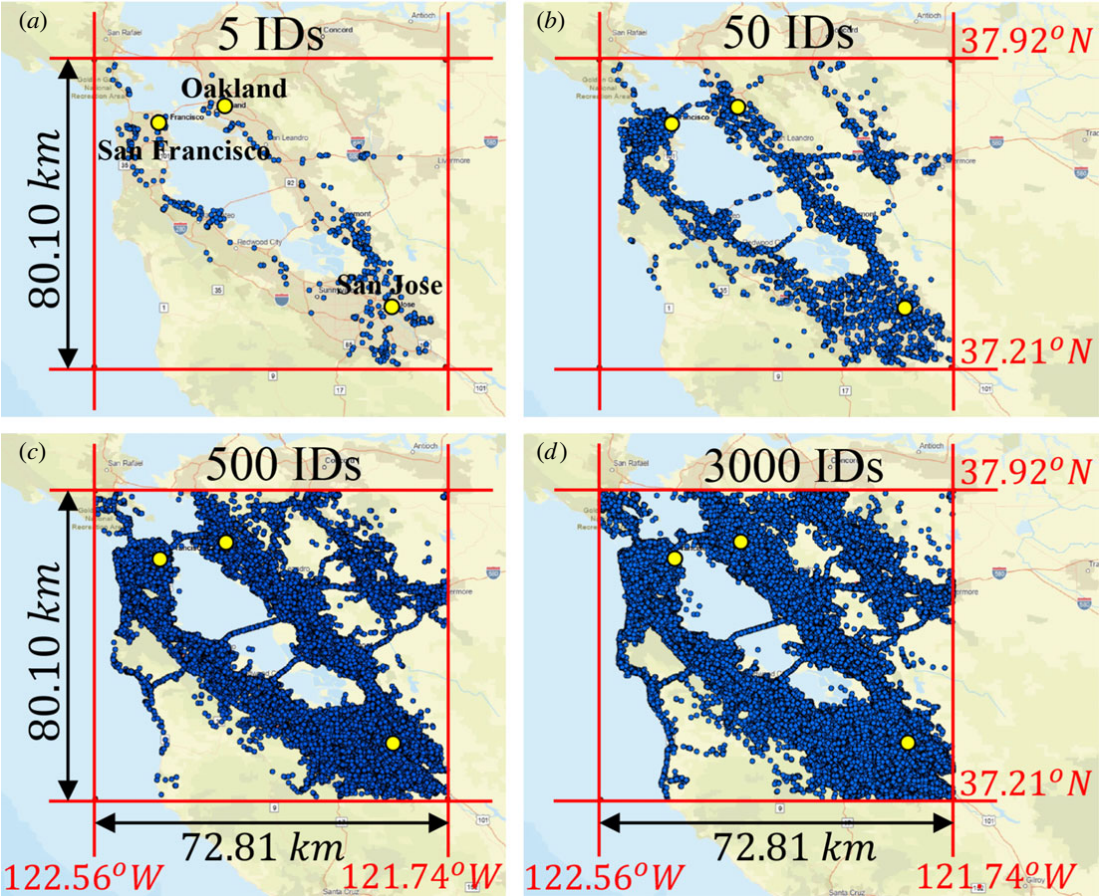
GPS locations of anonymous cell-phone users (IDs) in the San Francisco Area recorded during February and March 2021: (*a*) 5 IDs; (*b*) 50 IDs; (*c*) 500 IDs; (*d*) 3000 IDs; The yellow dots are nearby cities mentioned in (*a*).

**Figure 3.**
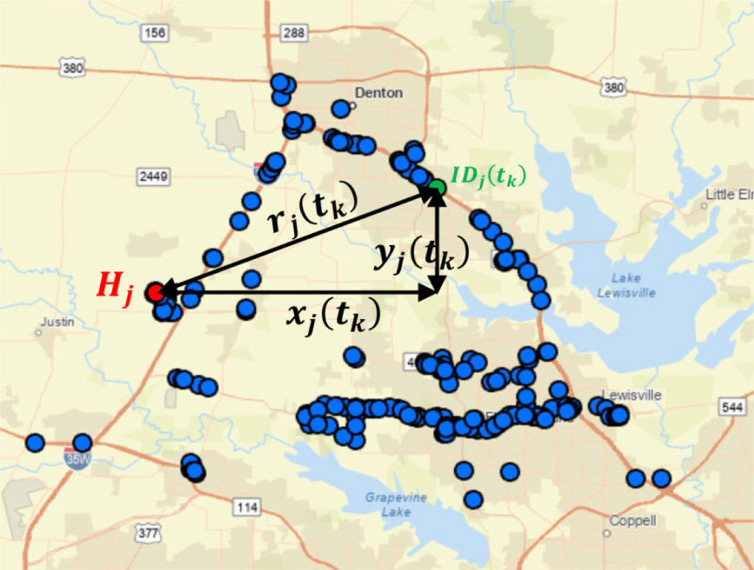
Displacement *r*_*j*_(*t*_*k*_) of ID_*j*_ at time *t*_*k*_ from its ‘home’ *H*_*j*_. In statistical mechanics and microrheology, the symbol Δ*r*_*j*_(*t*_*k*_) rather than *r*_*j*_(*t*_*k*_) is often used to express the distance of a probe from its ‘origin’. In this work, the symbol Δ is dropped since the displacement *r*_*j*_ is always measured from the fixed origin *H*_*j*_.

While the trace of the locations of each individual cell-phone ID shown in figures 1 and 2 is unique and every individual has a distinct purpose for generating the recorded trace, the collection of all traces of the anonymous cell-phone users in a given city is treated in this work as a random (stochastic) process. For each of the metroplexes examined in this work we processed longitude and latitude data (in degrees from the Greenwich meridian and from the equator) at various times for tens of thousands of anonymous cell-phone users (IDs). The data were processed and the origin of every ID (home) was identified by extracting the most frequent occupied location by the ID during the time interval 01.00 to 04.00. With reference to figure 3, the origin = ‘home’ of every ID is established; the east–west (E–W, longitude) and north–south (N–S, latitude) displacement of each ID_*j*_ at time *t*_*k*_ is calculated from [42]

2.1 $$x_{j}(t_{k})=\frac{\pi }{180}[{\mathrm{Long}}_{j}(t_{k})-\mathrm{Long}\;H_{j}(t_{k})]\cos\left(\frac{\pi }{180}\frac{\mathrm{Lat}\;H_{j}(t_{k})+{\mathrm{Lat}}_{j}(t_{k})}{2}\right)R_{E}$$

and

2.2 $$y_{j}(t_{k})=\frac{\pi }{180}[{\mathrm{Lat}}_{j}(t_{k})-\mathrm{Lat}\;H_{j}(t_{k})]R_{E},$$

where *R*_*E*_ = radius of Earth ≈ 6371 km and the displacement of ID_*j*_ at time *t*_*k*_ is $r_{j}(t_{k})=\sqrt{x_{j}^{2}(t_{k})+y_{j}^{2}(t_{k})}$ as shown in figure 3. For higher precision, one can use the *haversine formula*; yet the differences in the results from equations (2.1) and (2.2) are negligible given the relative small bounding areas of interest shown in figures 1 and 2. From equations (2.1) and (2.2), when ID_*j*_ moves to the east of its origin (home), *H*_*j*_, its displacement projection, *x*_*j*_, is negative; whereas, when it moves to the west of its home, *H*_*j*_, its displacement projection, *x*_*j*_ is positive. Similarly, when ID_*j*_ moves to the north of its home, *H*_*j*_, its displacement projection, *y*_*j*_, is positive; whereas, when it moves to the south of its home, *H*_*j*_, its displacement projection, *y*_*j*_, is negative. Accordingly, for every city, the GPS location data of *M* IDs are organized as shown in table 1. For every time *t*_*k*_ appearing in the first column of table 1 (say top of the hour), the location *x*_*j*_(*t*_*k*_), *y*_*j*_(*t*_*k*_) is the GPS location of ID_*j*_ recorded closer to that time *t*_*k*_.

**Table 1.** GPS location data of *M* cell-phone users (IDs) recorded closest to the top of the hour.

time	ID_1_			…	ID_*j*_			…	ID_*M*_		
*t*_1_	*x*_1_(*t*_1_)	*y*_1_(*t*_1_)	*r*_1_(*t*_1_)	…	*x*_*j*_(*t*_1_)	*y*_*j*_(*t*_1_)	*r*_*j*_(*t*_1_)	…	*x*_*M*_(*t*_1_)	*y*_*M*_(*t*_1_)	*r*_*M*_(*t*_1_)
*t*_2_	*x*_1_(*t*_2_)	*y*_1_(*t*_2_)	*r*_1_(*t*_2_)	…	*x*_*j*_(*t*_2_)	*y*_*j*_(*t*_2_)	*r*_*j*_(*t*_2_)	…	*x*_*M*_(*t*_2_)	*y*_*M*_(*t*_2_)	*r*_*M*_(*t*_2_)
⋮	⋮	⋮	⋮	…	⋮	⋮	⋮	⋮	…	⋮	⋮
*t*_*k*_	*x*_1_(*t*_*k*_)	*y*_1_(*t*_*k*_)	*r*_1_(*t*_*k*_)	…	*x*_*j*_(*t*_*k*_)	*y*_*j*_(*t*_*k*_)	*r*_*j*_(*t*_*k*_)	…	*x*_*M*_(*t*_*k*_)	*y*_*M*_(*t*_*k*_)	*r*_*M*_(*t*_*k*_)
⋮	⋮	⋮	⋮	…	⋮	⋮	⋮	⋮	…	⋮	⋮
*t*_*n*_	*x*_1_(*t*_*n*_)	*y*_1_(*t*_*n*_)	*r*_1_(*t*_*n*_)	…	*x*_*j*_(*t*_*n*_)	*y*_*j*_(*t*_*n*_)	*r*_*j*_(*t*_*n*_)	…	*x*_*M*_(*t*_*n*_)	*y*_*M*_(*t*_*n*_)	*r*_*M*_(*t*_*n*_)

**Figure 4.**
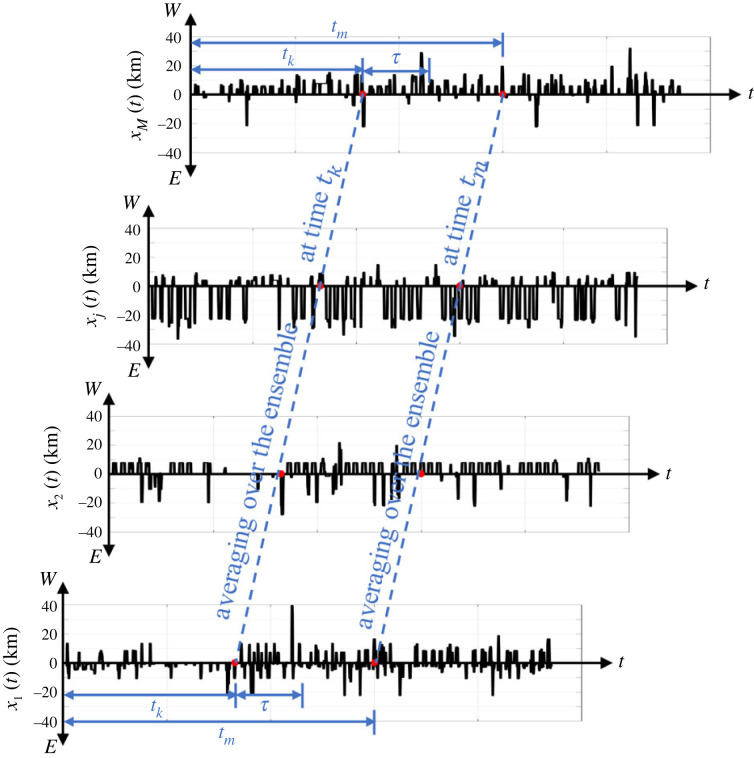
Ensemble time histories of the longitudinal (E–W) movement of IDs from the Dallas metroplex during February and March 2021.

Figure 4 plots time-histories of the E–W movement, *x*_*j*_(*t*), *j* ∈ {1, 2, … , *M*}, of selected IDs from the Dallas metroplex. Some IDs move frequently both to the east and to the west, other IDs move systematically to the east, other IDs move systematically to the west due to their daily routine (say travelling to their workplace). Accordingly, the time-averages from individual time-histories may differ drastically, therefore, the stochastic (random) process shown in figure 4 is clearly non-ergodic [43,44]. With reference to figure 4 for every time *t*_*k*_, we compute ensemble averages of the E–W movement 〈*x*(*t*_*k*_)〉 and the N–S movement 〈*y*(*t*_*k*_)〉 (mean values) for all the *M* available IDs

2.3a $$\left\langle x(t_{k})\rangle =\frac{1}{M}\sum_{\;j=1}^{M}x_{j}(t_{k}\right)$$

and

2.3b $$\langle y(t_{k})\rangle =\frac{1}{M}\sum_{\;j=1}^{M}y_{j}(t_{k}).$$

**Figure 5.**
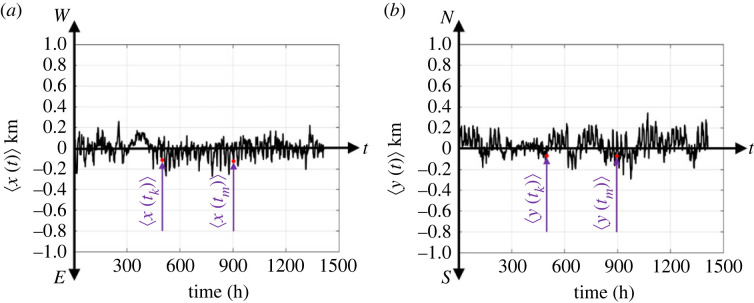
Time-histories of the mean values (ensemble averages) of the E–W movement 〈*x*(*t*_*k*_)〉 (*a*) and N–S movement 〈*y*(*t*_*k*_)〉 (*b*) of *M* = 13 000 IDs in the Dallas metroplex during February and March 2021.

Figure 5 plots the mean values (ensemble averages) 〈*x*(*t*_*k*_)〉 (left) and 〈*y*(*t*_*k*_)〉 (right) of *M* = 13 000 IDs in the Dallas metroplex for all times *t*_*k*_ from 1 February to 31 March 2021. Figure 5 uncovers that the time-histories of the mean values 〈*x*(*t*)〉 and 〈*y*(*t*)〉 fluctuate within only a couple of hundred metres.

Similarly, for every time *t*_*k*_ and a given time shift, *τ*, we compute the location (position) autocorrelation functions of the E–W movement 〈*x*(*t*_*k*_)*x*(*t*_*k*_ + *τ*)〉 and the N–S movement 〈*y*(*t*_*k*_)*y*(*t*_*k*_ + *τ*)〉 for all the *M* available IDs in a city (ensemble).

2.4a $$\left\langle x(t_{k})x(t_{k}+\tau )\rangle =\frac{1}{M}\sum_{\;j=1}^{M}x_{j}(t_{k})x_{j}(t_{k}+\tau \right)$$

and

2.4b $$\langle y(t_{k})y(t_{k}+\tau )\rangle =\frac{1}{M}\sum_{\;j=1}^{M}y_{j}(t_{k})y_{j}(t_{k}+\tau ).$$

**Figure 6.**
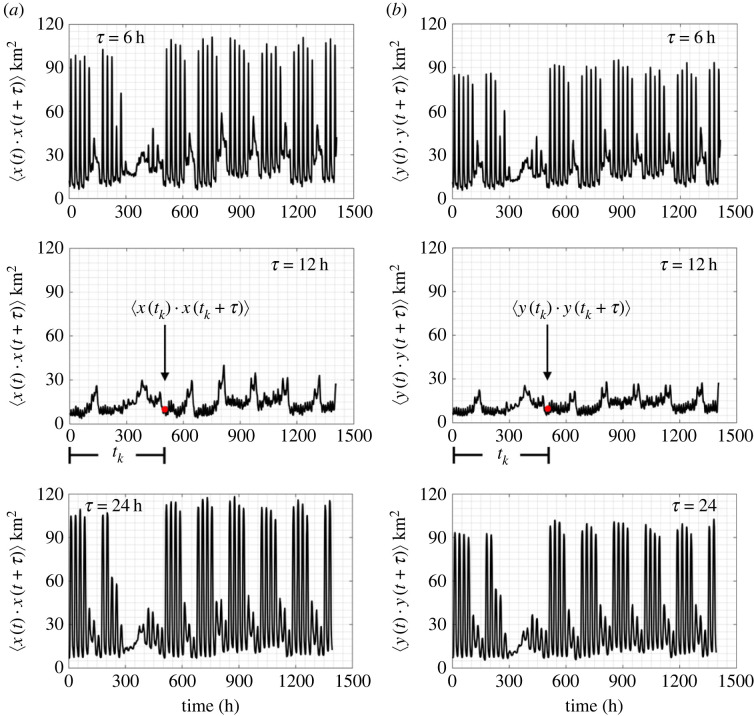
Time-histories of the location (position) autocorrelation functions (ensemble averages) of the E–W movement 〈*x*(*t*_*k*_) · *x*(*t*_*k*_ + *τ*)〉 (*a*) and the N–S movement 〈*y*(*t*_*k*_) · *y*(*t*_*k*_ + *τ*)〉 (*b*) of the *M* = 13 000 IDs in the Dallas metroplex during February and March 2021 for three different values of the time shift *τ* = 6 h, 12 h and 24 h.

Figure 6 plots the location (position) autocorrelation functions (ensemble averages) 〈*x*(*t*_*k*_)*x*(*t*_*k*_ + *τ*)〉 (*a*) and 〈*y*(*t*_*k*_)*y*(*t*_*k*_ + *τ*)〉 (*b*) of *M* = 13 000 IDs in the Dallas metroplex for all times *t*_*k*_ and time shifts *τ* = 6 h, 12 h and 24 h. Clearly, for *τ* = 24 h, the location autocorrelation functions shown in figure 6 show a stronger correlation than when *τ* = 12 h, since after 24 h the cell-phone users are likely to be at the same location that they were at the day before. Accordingly, while the mean values of the locations (positions) shown in figure 5 fluctuate within only a couple of hundred metres, the strong fluctuations over time of the position autocorrelation functions shown in figure 6 indicate that the stochastic (random) process schematically illustrated in figure 4 does not even satisfy the notion of a weakly stationary process [43,44]. Accordingly, in this study, we work with ensemble averages (not time averages).

## 3. Theory

The analysis framework that we develop herein to construct a mechanical analogue for cities is inspired from major advances in microrheology in the mid- to late 1990s [27–37] which make possible the extraction of the bulk mechanical properties of complex viscoelastic materials by monitoring the thermally driven Brownian motion of probe microspheres suspended within the viscoelastic material.

The phenomenon of Brownian motion was first explained in Einstein’s celebrated 1905 paper [45] which examined the long-term response of Brownian microspheres with mass *m* and radius *R* suspended in a memoryless Newtonian fluid with viscosity *η*. Einstein’s theory of Brownian motion predicts the long-term expression of the MSD, 〈*r*^2^ (*t*)〉 = MSD, of the randomly moving microspheres (diffusive regime)

3.1 $$\langle r^{2}(t)\rangle =\frac{1}{M}\sum_{\;j=1}^{M}r_{j}^{2}(t)=2NDt=\frac{NK_{B}T}{3\pi R}\frac{t}{\eta },$$

where *M* is the number of suspended microspheres, while, *r*_*j*_ (*t*) is the distance of microsphere *j* at time *t* from some origin. In equation (3.1), *N* ∈ {1, 2, 3} is the number of spatial dimensions, *K*_*B*_ is Boltzmann’s constant, *T* is the equilibrium temperature of the Newtonian fluid with viscosity *η* within which the *M* Brownian microspheres are immersed and *D* = *K*_*B*_ *T*/6*πRη* is the time-independent diffusion coefficient. Equation (3.1) derived by Einstein in 1905, in association with Stokes’ result that the drag force on a slowly moving sphere with velocity *v*, is 6*πRηv* [46], is of central interest in statistical mechanics since it relates the thermally driven ensemble average 〈*r*^2^ (*t*)〉 from a stochastic process to the deterministic emergent property of the Newtonian fluid—that is its viscosity, *η*. The time-derivative of equation (3.1) gives

3.2 $$\frac{d\langle r^{2}(t)\rangle }{dt}=\frac{2}{M}\sum_{\;j=1}^{M}|r_{j}(t)\frac{dr_{j}(t)}{dt}|=2ND=\frac{NK_{B}T}{3\pi R\eta }.$$

The reader recognizes that the right-hand side term of equation (3.2) is a constant =*NK*_*B*_ *T*/3*πRη* = 2*ND*; whereas, the left-hand side $(2/M)\sum_{\;j=1}^{M}|r_{j}(t)(dr_{j}(t)/dt)|$ is zero when *t* = 0, since $r_{j}(0)=0,\;\forall j\in \{1,2,\ldots ,M\}$. This inconsistency emerges because equation (3.1) is valid only in the longterm (diffusive regime at large times). At short timescales, when *t* < *m*/6*πRη* = *τ*, the Brownian motion of suspended particles is influenced by the inertia of the particle and the surrounding fluid (ballistic regime) and Einstein’s 1905 ‘long-term’ result offered by equation (3.1) was extended for all timescales by Uhlenbeck & Ornstein [47].

3.3 $$\left\langle r^{2}(t)\rangle =\frac{NK_{B}T}{3\pi R\eta }[t-\tau (1-e^{-t/\tau })\right]$$

Equation (3.3) yields that at *t* = 0, d〈*r*^2^ (*t*)〉/d*t* = 0, which is in agreement with the left-hand side of equation (3.2) when *t* = 0.

### 3.1. Generalization for Brownian motion within non-Newtonian fluids

In their seminal paper, Mason & Weitz [27] employed dynamic light scattering to measure the MSD 〈*r*^2^(*t*)〉 of probe Brownian particles immersed in a linear viscoelastic fluid and related it to the complex dynamic modulus, $G_{\mathrm{ve}}(s)$, of the viscoelastic fluid within which the probe particles are immersed. Given the viscoelastic behaviour of the complex fluid, the motion of a probe Brownian particle is described by the generalized Langevin equation [48,49]

3.4 $$m\frac{dv(t)}{dt}=-\int_{0^{-}}^{t}\zeta (t-\xi )v(\xi )\;d\xi +f_{R}(t),$$

where *m* is the mass of the Brownian particle, *v*(*t*) is the particle velocity and *f*_*R*_ (*t*) are the random forces acting on the particle from the collisions of the fluid molecules on the Brownian particle. The integral in equation (3.4) represents the drag force on the particle as it moves through the viscoelastic fluid and accounts for the fading memory of this drag due to the elasticity of the viscoelastic fluid. Upon transforming equation (3.4) in the Laplace domain, Mason & Weitz [27] reached the following result for the Laplace transform of the MSD:

3.5 $$\langle r^{2}(s)\rangle =\int_{0}^{\infty }\langle r^{2}(t)\rangle \;e^{-st}\;dt=\frac{NK_{B}T}{3\pi R}\frac{1}{s(G_{\mathrm{ve}}(s)+(m/6\pi R)s^{2})},$$

where *s* is the Laplace variable. In deriving equation (3.5) a key assumption was adopted—that the Stokes drag coefficient on a sphere moving slowly in a memoryless, Newtonian viscous fluid, *ζ* = 6*πRη* [46], can be generalized to relate the impedance of the Brownian particle, $Z(s)=L\{\zeta (t)\}=\int_{0^{-}}^{\infty }\zeta (t)\;e^{-st}\;dt$ to the complex dynamic viscosity of the viscoelastic material, $\eta_{\mathrm{ve}}(s)=G_{\mathrm{ve}}(s)/s$. Accordingly, Mason & Weitz [27] made the physically motivated assumption that

3.6 $$Z(s)=\int_{0}^{\infty }\zeta (t)\;e^{-st}\;dt=6\pi R\eta_{\mathrm{ve}}(s)=6\pi R\frac{G_{\mathrm{ve}}(s)}{s}$$

In a recent publication, Makris [50] showed that the time response function *J*(*t*) = (1/*η*)[*t* − *τ*(1 − e^−*t*/*τ*^)] appearing on the right-hand side of equation (3.3) is the creep compliance (retardation function) of a linear network where a dashpot with viscosity *η* is connected in parallel with an inerter with distributed inertance *m*_*R*_ = *m*/6*πR*. Building on the work of [47,51] in association with the work of Mason & Weitz [27], Makris [50] uncovered a viscous-viscoelastic correspondence principle for Brownian motion which states that the MSD 〈*r*^2^ (*t*)〉 of Brownian particles (microspheres) with mass *m* and radius *R* suspended in some linear, isotropic viscoelastic material when subjected to the random forces from the collisions of the molecules of the viscoelastic material, is

3.7 $$\langle r^{2}(t)\rangle =\frac{NK_{B}T}{3\pi R}J(t),$$

where *J*(*t*) is the creep compliance (strain history due to a unit-step stress) of a viscoelastic network that is a parallel connection of the linear viscoelastic material within which the Brownian particles are immersed and an inerter with distributed inertance *m*_*R*_ = *m*/6*πR*. Equation (3.7) also holds for the case where the density of the fluid surrounding the Brownian microparticles is appreciable [52] and in this case, in addition to the Stokes viscous drag, the Brownian motion of the immersed microparticles develops the Boussinesq hydrodynamic memory [53].

Equation (3.7), which is the equivalent of equation (3.5) in the time domain, generalizes the main result of equation (3.1) and relates the thermally driven ensemble average 〈*r*^2^ (*t*)〉 = MSD from a stochastic (random) process to the deterministic creep compliance (retardation function), *J*(*t*) of a mechanical network. In this work, we build upon equation (3.7) in an effort to propose a corresponding relation for cities,

3.8 $$\langle r^{2}(t)\rangle =WJ(t),$$

where 〈*r*^2^ (*t*)〉 is the MSD of cell-phone users as computed by averaging over a city ensemble of GPS location traces, *J*(*t*) is the creep compliance (displacement history due to a unit-step force) of a mechanical model to be identified and *W* is a proportionality constant having the units of energy (force × length).

**Figure 7.**
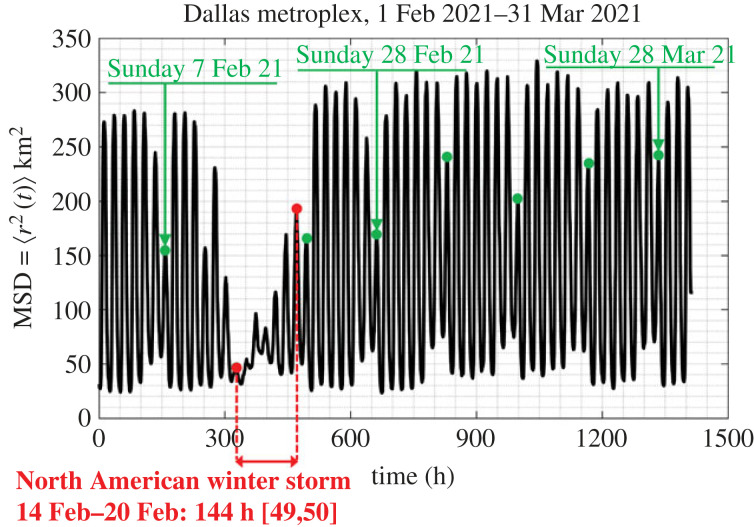
Time history of the mean-square displacement $=\langle r^{2}(t)\rangle =(1/M)\sum_{\;j=1}^{M}r_{j}^{2}(t)$ (ensemble average) of *M* = 13 000 IDs in the Dallas metroplex during February and March 2021. Dots (in green) atop the suppressed spikes indicate Sundays.

The motion of people in a city as recorded with their GPS locations happens in a matterless environment (the environment of a city which does not exhibit mechanical elasticity, dissipation or inertia); while it is driven by conceptual incentives; therefore, we do not have a Langevin equation to describe the motion of people in cities as we have equation (3.4) that describes the random motion of Brownian particles immersed in some linear, isotropic viscoelastic material. Furthermore, the statistical analysis of the GPS location data processed in this study yields that ensemble averages as illustrated in figure 4 do not even support the notion of a weak stationary random process [43,44]. Nevertheless, in view of the overall phenomenological similarities between the motion of people in a city (figures 1 and 2) and the motion of probe Brownian microparticles in a viscoelastic material in association with the remarkable success of equations (3.5) or (3.7) in microrheology, we adopt equation (3.8) in an effort to develop a mechanical model for cities with potential engineering significance. Figure 7 plots the time history of the MSD = 〈*r*^2^ (*t*)〉

**Figure 8.**
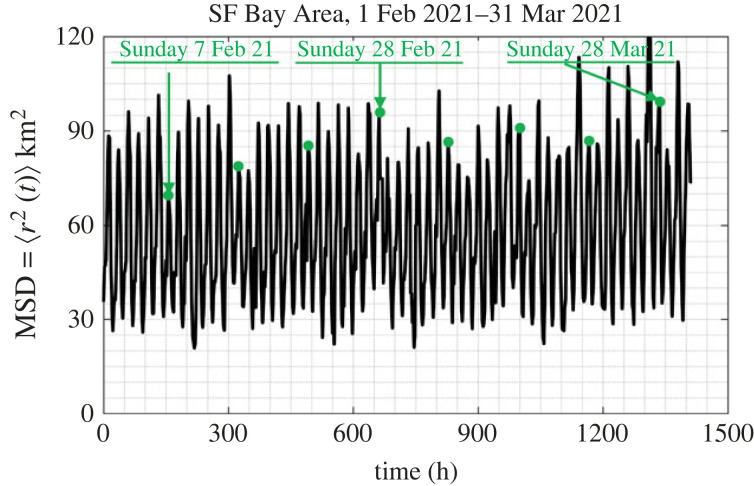
Time history of the mean-square displacement $=\langle r^{2}(t)\rangle =(1/M)\sum_{\;j=1}^{M}r_{j}^{2}(t)$ (ensemble average) of *M* = 3400 IDs in the San Francisco Bay Area during February and March 2021. Dots (in green) atop the suppressed spikes indicate Sundays.

3.9 $$\left\langle r^{2}(t)\rangle =\frac{1}{M}\sum_{\;j=1}^{M}r_{j}^{2}(t)=\frac{1}{M}\sum_{\;j=1}^{M}x_{j}^{2}(t)+y_{j}^{2}(t\right)$$

of *M* = 13 000 cell-phone users (IDs) in the Dallas metroplex during February and March 2021; while, figure 8 plots the time history of 〈*r*^2^ (*t*)〉 of *M* = 3400 cell-phone users (IDs) in the San Francisco Bay Area during the same time.

Figure 7 reveals that the MSD for the Dallas metroplex exhibits a remarkable periodicity with people moving more during the weekdays and less on Sundays which are marked with green dots. At the same time, less people are staying at home during the weekends than during the weekdays. The MSD from the Dallas metroplex exhibits a notable suppression during the major North American winter storm that happened during the third week of February 2021 and ended sometime towards the end of that week before Sunday, 21 February 2021. Over 4 million people lost power due to the storm which resulted to a state-wide power crisis in Texas causing major damage to the Texas power grid [54,55]. Regardless of its unprecedented intensity and resulting disruption from the winter storm, figure 7 reveals that immediately after the cold winter storm, the city of Dallas resumes normal activity with the MSD of its citizens reverting to its normal oscillation pattern as recorded before the storm, suggesting that the Dallas metroplex exhibits a great degree of engineering resilience [38].

The MSD from the San Francisco (SF) Bay Area for the same period (1 February–31 March 2021) is less ordered that the MSD from the Dallas metroplex; nevertheless the same pattern of less people staying at home during the weekends than during the weekdays is observed. Furthermore, the average MSD from the SF Bay Area covers less area (on an average 60 km^2^) when compared with 165 km^2^ from the Dallas metroplex. Both cities ‘never sleep’ since the MSDs from both the Dallas metroplex (figure 7) and from the San Francisco Bay Area (figure 8) reach a bottom at approximately 30 km^2^.

The smaller values of the average MSD of the SF Bay Area when compared with that from the Dallas metroplex is partly due to the presence of the water boundaries (Pacific Ocean and SF Bay) in association with the presence of the Santa Cruz mountains to the southwest (figure 2). At the same time, the SF Bay Area is served with pronounced traffic corridors which are connected with the major bridges over the Bay Area leading to different traffic patterns than these of the landlocked Dallas metroplex. Nevertheless, the recorded MSD for the San Francisco Bay Area shown in figure 8 and its satisfactorily match with the proposed amplitude-modulation model shown in figure 13 suggest that our mechanical model may be also promising for cities with pronounced traffic corridors and traffic patterns other than these of the Dallas metroplex. The cell-phone mobility data that we have access to at this time go back to 2019 only; and for this 4-year period, there has not been any natural-hazard (earthquake) that struck the San Francisco Bay Area to confirm the predictions of the proposed mechanical model for such cities.

## 4. Candidate mechanical analogues for cities

Figures 7 and 8 show that the MSD, 〈*r*^2^ (*t*)〉, of cell-phone users as computed by averaging over an ensemble of citizens from major urban areas, is essentially an elevated cosine with a daily period where the amplitude of the cosine oscillation is modulated with a weekly period. Accordingly in view of equation (3.8), in this section we are in search of mechanical networks that their creep compliance, *J*(*t*) (displacement history due to a unit-step force) can approximate the time history of the MSD, 〈*r*^2^ (*t*)〉 as computed from ensemble averages and shown in figures 7 and 8.

Given that the MSD, 〈*r*^2^ (*t*)〉, shown in figures 7 and 8 are bounded functions, in association with equation (3.8) we are in search of a solid-like network so that its creep compliance, *J*(*t*), is a bounded time response function. Furthermore, the perpetual undamped oscillations of the computed 〈*r*^2^ (*t*)〉 with no visible rise-time imply that the candidate mechanical network shall be undamped. Finally, given that the cosine function shown in figures 7 and 8 is elevated above the horizontal axis, the candidate network needs to yield a creep compliance function that is more elaborate than the elementary inertoelastic solid (the spring-inerter parallel connection) [56,57].

### 4.1. The three-parameter inertoelastic solid

**Figure 9.**
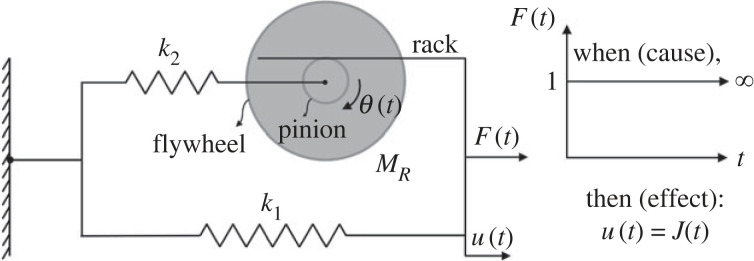
The three-parameter introelastic solid which is a parallel connection of an introelastic fluid (spring-inerter in-series connection) with an elastic spring with stiffness *k*_1_.

The simplest mechanical network that meets the above requirements is a parallel connection of an elastic spring with an inertoelastic fluid (spring-inerter in-series connection) as shown in figure 9. The total force *F*(*t*) = *F*_1_(*t*) + *F*_2_(*t*) from the linear network shown in figure 9 is the summation of the force output from the linear spring with elastic constant *k*_1_,

4.1 $$F_{1}(t)=k_{1}u(t)$$

and the force output from the inertoelastic fluid [56]

4.2 $$F_{2}(t)+\frac{1}{\omega_{R2}^{2}}\frac{d^{2}F_{2}(t)}{dt^{2}}=M_{R}\frac{d^{2}u(t)}{dt^{2}},$$

where *M*_*R*_ is the inertance of the inerter with units of mass [*M*] and $\omega_{R2}^{2}=k_{2}/M_{R}$ is the rotational frequency of the inertoelastic fluid. The summation of equations (4.1) and (4.2) together with the second time-derivative of equation (4.1) yields the constitutive equation of the three-parameter inertoelastic solid shown in figure 9.

4.3 $$F(t)+\frac{1}{\omega_{R2}^{2}}\frac{d^{2}F(t)}{dt^{2}}=k_{1}\left[u(t)+\left(\frac{1}{\omega_{R1}^{2}}+\frac{1}{\omega_{R2}^{2}}\right)\frac{d^{2}u(t)}{dt^{2}}\right],$$

where $\omega_{R1}^{2}=k_{1}/M_{R}$. Upon replacing

4.4 $$\frac{1}{\omega_{R1}^{2}}+\frac{1}{\omega_{R2}^{2}}=\frac{1}{\omega_{R}^{2}}\Rightarrow \omega_{R}^{2}=\frac{\omega_{R1}^{2}\omega_{R2}^{2}}{\omega_{R1}^{2}+\omega_{R2}^{2}}=\frac{\omega_{R2}^{2}}{1+(\omega_{R2}^{2}/\omega_{R1}^{2})}$$

the Laplace transform of equation (4.3) gives $u(s)=L\{u(t)\}=\int_{0}^{\infty }u(t)\;e^{-st}\;dt=H(s)F(s)$, where H(s) is the complex dynamic flexibility of the linear network shown in figure 9.

4.5 $$H(s)=\frac{u(s)}{F(s)}=\frac{1}{k_{1}}\frac{1+(s^{2}/\omega_{R2}^{2})}{1+(s^{2}/\omega_{R}^{2})}=\frac{1}{k_{1}}\left[\frac{\omega_{R}^{2}}{s^{2}+\omega_{R}^{2}}+\frac{\omega_{R}^{2}}{\omega_{R2}^{2}}\frac{s^{2}}{s^{2}+\omega_{R}^{2}}\right].$$

The Laplace transform of the creep compliance, *J*(*t*) is the complex creep function $C(s)=L\{J(t)\}=\int_{0}^{\infty }J(t)\;e^{-st}\;dt=H(s)/s$, [58–61]. Upon dividing equation (4.5) with the Laplace variable *s*, we obtain

4.6 $$\begin{array}{rl} C(s) & =\frac{H(s)}{s}=\frac{1}{k_{1}}\left[\frac{1}{s}\frac{\omega_{R}^{2}}{s^{2}+\omega_{R}^{2}}+\frac{\omega_{R}^{2}}{\omega_{R2}^{2}}\frac{s}{s^{2}+\omega_{R}^{2}}\right]=\frac{1}{k_{1}}\left[\frac{1}{s}-\frac{s}{s^{2}+\omega_{R}^{2}}+\frac{\omega_{R}^{2}}{\omega_{R2}^{2}}\frac{s}{s^{2}+\omega_{R}^{2}}\right]. \end{array}$$

The inverse Laplace transform of equation (4.6) offers the creep compliance of the three-parameter inertoelastic solid shown in figure 9.

4.7 $$J(t)=L^{-1}\{C(s)\}=\frac{1}{k_{1}}\left[U(t-0)-\frac{\beta }{1+\beta }\cos(\omega_{R}t)\right],$$

where *U*(*t* − 0) is the Heaviside unit-step function at the time origin [62] and parameter $\beta =\omega_{R2}^{2}/\omega_{R1}^{2}=k_{2}/k_{1}$.

The expression of the creep compliance, *J*(*t*) offered by equation (4.7) is an elevated cosine function with frequency $\omega_{R}=\omega_{R2}/\sqrt{1+\beta }=2\pi /24\;h=\pi /12\;\mathrm{rad}\;h^{-1}$. Substitution of equation (4.7) into equation (3.8), which is the central equation for this study, the elementary three-parameter inertoelastic solid mechanical analogue predicts that

4.8 $$\langle r^{2}(t)\rangle =WJ(t)=\frac{W}{k_{1}}\left[U(t-0)-\frac{\beta }{1+\beta }\cos(\omega_{R}t)\right],$$

where the proportionality constant *W*/*k*_1_ = *A*_0_ has units of area (km^2^). Accordingly, given that $\omega_{R}=\omega_{R2}/\sqrt{1+\beta }$ is the daily frequency (*ω*_*R*_ = *π*/12 rad h^−1^), only two parameters of the three-parameter inertoelastic solid need to be calibrated from the recorded GPS location data; *A*_0_ = *W*/*k*_1_, and $\beta =k_{2}/k_{1}=\omega_{R2}^{2}/\omega_{R1}^{2}$.

**Figure 10.**
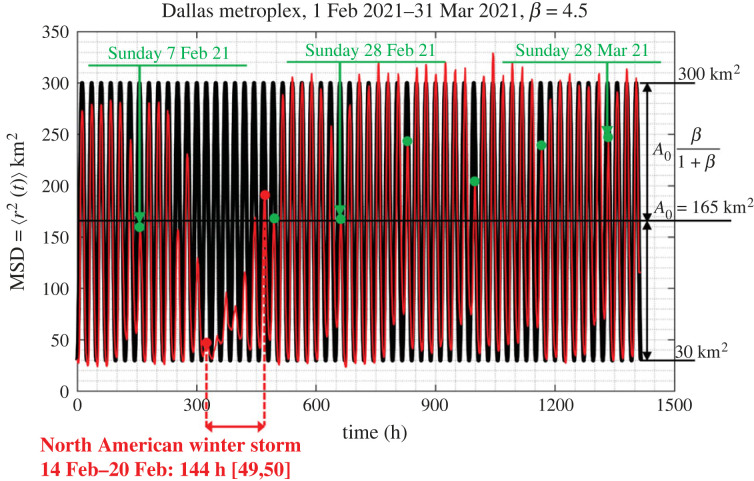
Comparison of the result of equation (4.8), 〈*r*^2^ (*t*)〉 = *WJ*(*t*) with the recorded mean-square displacement, 〈*r*^2^ (*t*)〉 from the Dallas metroplex during February and March 2021.

**Figure 11.**
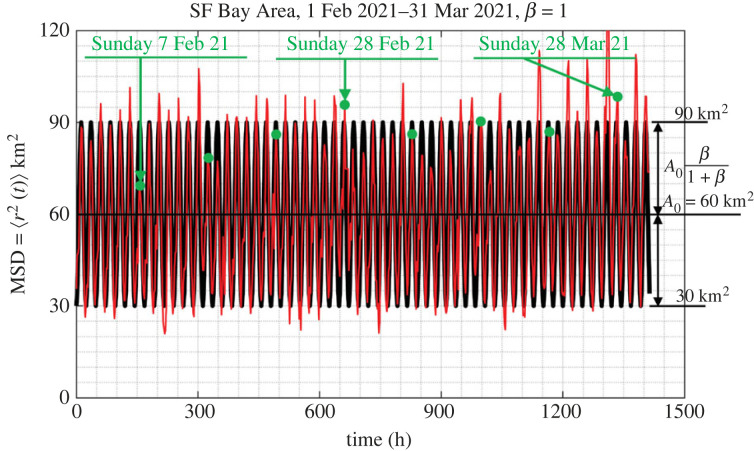
Comparison of the result of equation (4.8), 〈*r*^2^ (*t*)〉 = *WJ*(*t*) with the recorded mean-square displacement, 〈*r*^2^ (*t*)〉 from the San Francisco Bay Area during February and March 2021.

Figures 10 and 11 compare the predictions of the calibrated three-parameter inertoelastic solid shown in figure 9 against the recorded MSD = 〈*r*^2^ (*t*)〉 of *M* = 13 000 IDs in the Dallas metroplex (figure 10) and the resulting MSD = 〈*r*^2^ (*t*)〉 of 3400 IDs in the San Francisco Bay Area (figure 11) during February and March 2021. The MSD, 〈*r*^2^ (*t*)〉, shown in figures 10 and 11 as computed by averaging over a city ensemble of GPS location traces suggests that the coefficient *β*/(1 + *β*) of the cosine function in equation (4.7) or (4.8) is the average normalized amplitude of the daily oscillations of 〈*r*^2^ (*t*)〉. With reference to figure 10 for the Dallas metroplex, β/(1+β)=(1/2)((300−30)/165)≈0.8181⟹β=4.5 with *A*_0_ = *W*/*k*_1_ = 165 km^2^; whereas, for the San Francisco Bay Area β/(1+β)=(1/2)((90−30)/60)=0.5⟹β=1.0 with *A*_0_ = *W*/*k*_1_ = 60 km^2^.

### 4.2. An amplitude-modulation model

The time history of the MSD 〈*r*^2^ (*t*)〉 of the Dallas metroplex IDs shown in figure 7 exhibits a distinguishable weekly modulation (message signal) in addition to the daily oscillations (carrier wave), *C*(*t*) = *C*cos (*ω*_*R*_ *t*) with *ω*_*R*_ = 2*π*/24 h = *π*/12 rad h^−1^. By expressing the weekly modulating message signal [63,64]

4.9 $$s(t)=\gamma C\sin\left(\frac{\omega_{R}}{7}t\right)$$

an improved mathematical expression to equation (4.8) that better approximates the Dallas metroplex MSD shown in figure 7 is

4.10 $$\langle r^{2}(t)\rangle =A_{0}\left[U(t-0)-C\cos(\omega_{R}t)\left[1+\gamma \sin\left(\frac{\omega_{R}}{7}t\right)\right]\right],$$

where *C* and *γ* are dimensionless model parameters. Upon using the trigonometric identity $\cos(\omega_{R}t)\sin((\omega_{R}/7)t)=\frac{1}{2}[\sin(\frac{8}{7}\omega_{R}t)-\sin(\frac{6}{7}\omega_{R}t)]$, equation (4.11) assumes the form

**Figure 12.**
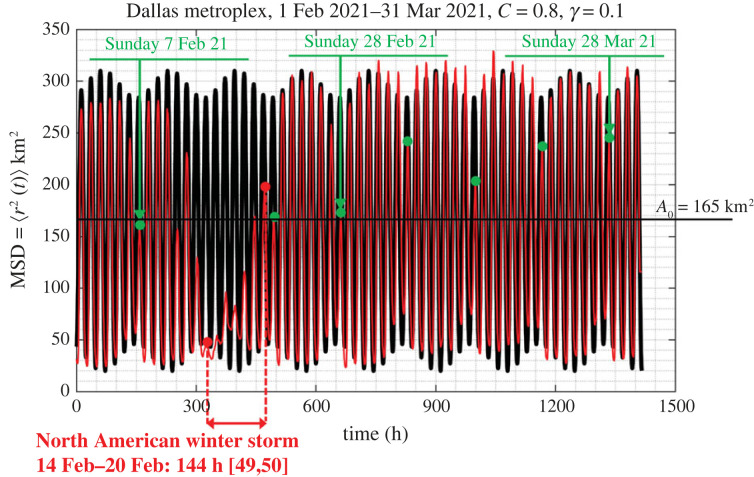
Comparison of the amplitude-modulation expression given by equation (4.11) with the recorded mean-square displacement 〈*r*^2^ (*t*)〉 of equation (4.11) *M* = 13 000 IDs from the Dallas metroplex during February and March 2021.

**Figure 13.**
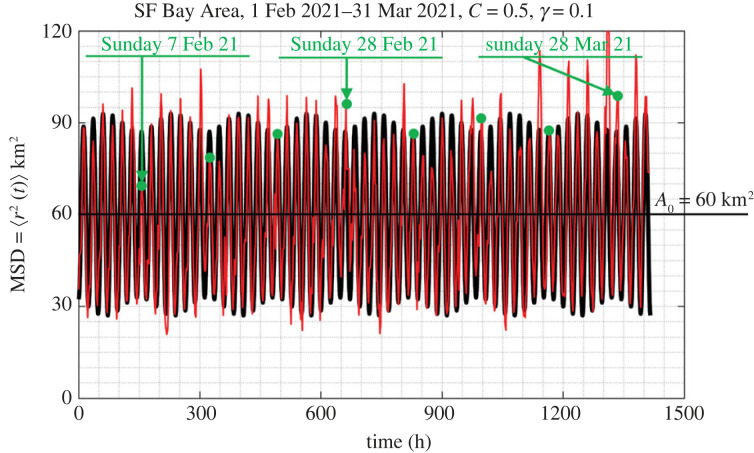
Comparison of the amplitude-modulation expression given by equation (4.11) with the recorded mean-square displacement 〈*r*^2^ (*t*)〉 of *M* = 3400 IDs from the San Francisco Bay Area during February and March 2021.

4.11 $$\begin{array}{rl} \left\langle r^{2}(t)\right\rangle & =WJ(t)=A_{0}\left[U(t-0)-C\left[\cos(\omega_{R}t)+\frac{\gamma }{2}\left(\sin\left(\frac{8}{7}\omega_{R}t\right)-\sin\left(\frac{6}{7}\omega_{R}t\right)\right)\right]\right], \end{array}$$

where *A*_0_ = *W*/*k* is a proportionality constant with units [*L*]^2^ that relates the MSD to a normalized creep compliance (see also equation (4.8)). Parameter *k* has units of stiffness (force/length). For the mathematical model offered by equation (4.11), we do not have a mechanical model that consists of a collection of springs and inerters as is the three-parameter inertoelastic model shown in figure 9. The model expressed by equations (4.10) or (4.11) was merely constructed from experience in manipulating signals in radio communications [63,64]. Figures 12 and 13 compare the result of equation (4.11) against the recorded MSD = 〈*r*^2^ (*t*)〉 in the Dallas metroplex (figure 12) and the recorded MSD = 〈*r*^2^ (*t*)〉 in the San Francisco Bay Area (figure 13) during February and March 2021. The amplitude-modulation expression for 〈*r*^2^ (*t*)〉 given by equation (4.11) captures the observed behaviour that more activity happens during the weekdays, while less people are staying at home during the weekends than during the weekdays.

## 5. Response of the candidate models to a rectangular pulse with duration *T*_*e*_

We return now to the mechanical model depicted in figure 9 which is the simplest mechanical analogue for a city since its creep compliance *J*(*t*) given by equation (4.7) offers according to equation (3.8) a MSD 〈*r*^2^ (*t*)〉 that matches to a satisfactory extent the recorded MSD as results from the GPS location data of several thousand users (figures 10 and 11).

The main motivation of this study is to develop quantitative models for cities that have some predictive capability. Our response analysis starts with an elementary rectangular force pulse of amplitude *F*_*o*_ and duration *T*_*e*_ which is expressed as

5.1 $$F(t)=F_{0}[U(t-0)-U(t-T_{e})],$$

where *U*(*t* − *ξ*) is the unit-amplitude Heaviside step function that initiates at time *t* = *ξ* [62]. The rectangular pulse with duration *T*_*e*_ expressed by equation (5.1) is suitable to express shocks from natural hazards (earthquakes, hurricanes and cold storms) which start and end abruptly and are system-wide [65]. By contrast, Gaussian functions with long tails are less suitable to approximate natural hazards which impact cities suddenly with little or zero loading build-up time. Pandemics and economic crises are stressing cities during larger timescales during which the city may develop mechanisms to readjust [13,66]. Therefore, for such prolonged crisis, different loading patterns than the rectangular pulse described with equation (5.1) may be more suitable.

The response of the three-parameter inertoelastic model shown in figure 9, which is described with the constitutive equation (4.3), to the forcing function given by equation (5.1) can be computed with equation (4.5), which gives u(s)=H(s)F(s), in which *F*(*s*) is the Laplace transform of *F*(*t*) given by equation (5.1)

5.2 $$F(s)=\int_{0}^{\infty }F(t)\;e^{-st}\;dt=F_{0}\int_{0}^{T_{e}}\;e^{-st}\;dt=F_{0}\left(\frac{1}{s}-\frac{1}{s}\;e^{-st}\right)$$

and H(s) is the complex dynamic flexibility, given by equation (4.5). Accordingly, under a rectangular pulse-load

5.3 $$u(s)=F_{0}\left[\frac{1}{s}H(s)-\frac{1}{s}H(s)\;e^{-sT_{e}}\right].$$

### 5.1. Response of the three-parameter inertoelastic solid to a rectangular pulse

Recognizing that H(s)/s=C(s) is the complex creep function expressed by equation (4.6) and after expanding the first term in the bracket of the right-hand side of equation (4.5) in partial fractions

5.4 $$\frac{1}{s}\frac{\omega_{R}^{2}}{s^{2}+\omega_{R}^{2}}=\frac{1}{s}-\frac{s}{s^{2}+\omega_{R}^{2}}$$

the inverse Laplace transform of equation (5.3) gives

5.5 $$u(t)=F_{0}\left[J(t)-\frac{1}{k_{1}}L^{-}1\left(\frac{1}{s}\;e^{-sT_{e}}-\frac{s}{s^{2}+\omega_{R}^{2}}\;e^{-sT_{e}}\right)-\frac{1}{k_{1}}\frac{\omega_{R}^{2}}{\omega_{R2}^{2}}\cos[\omega_{R}(t-T_{e})]\right].$$

Upon replacing in equation (5.5) the expression of *J*(*t*) given by equation (4.7) and after performing the remaining inverse Laplace transforms appearing in equation (5.5), the response of the three-parameter inertoelastic solid shown in figure 9 when subjected to the rectangular pulse loading given by equation (5.1) is

5.6 $$u(t)=\frac{F_{0}}{k_{1}}\left[U(t-0)-U(t-T_{e})-\frac{\beta }{1+\beta }\cos(\omega_{R}t)+\frac{\beta }{1+\beta }\cos[\omega_{R}(t-T_{e})]\right].$$

**Figure 14.**
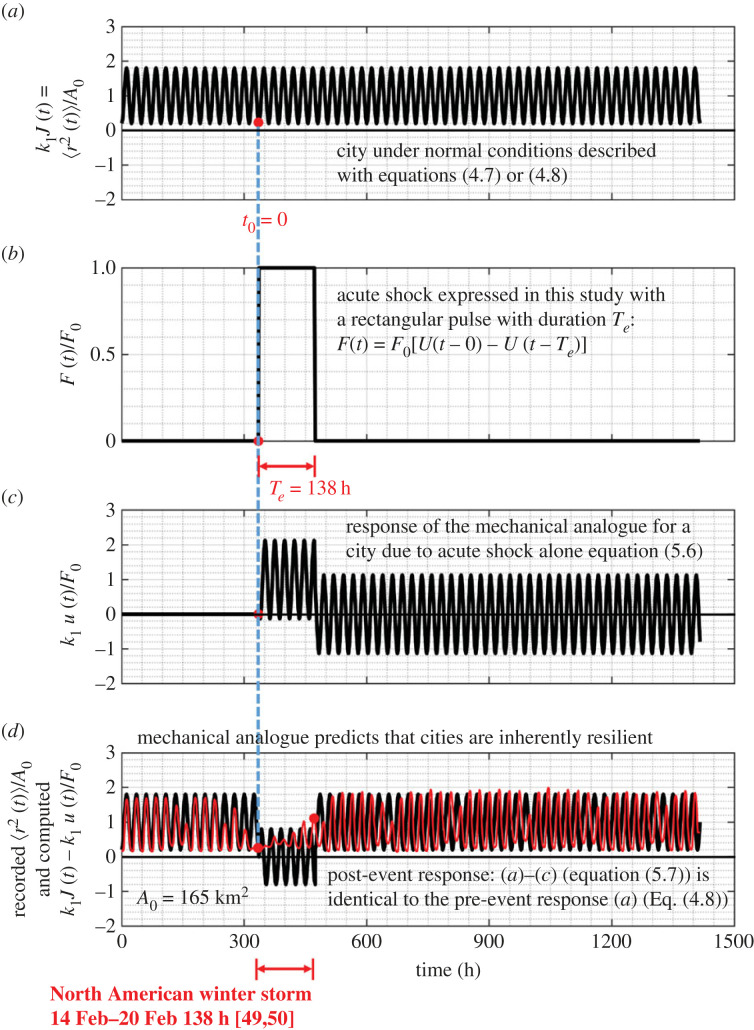
(*a*) Normalized behaviour of a city under normal conditions as given by equations (4.7) or (4.8). (*b*) Acute shock on a city expressed with a rectangular pulse with duration *T*_*e*_ = 138 h that is not an integer multiple of a day = 24 h. (c) Normalized response of a city to a rectangular pulse alone with duration *T*_*e*_ = 138 h. (*d*) Pre- and post-event response of a city ((*a*–*c*), solid dark lines) which is identical to the pre-event response of the city (*a*). The thin red line is the normalized MSD of the Dallas metroplex prior, during and after the North American winter storm during the third week of February 2021 as computed from the recorded GPS location data.

Clearly, when the duration of the acute shock (rectangular pulse) is an integer multiple of a day (*T*_*e*_ = *n*(24 h) = *n*(24 h/2*π*)2*π* = *n*(2*π*/*ω*_*R*_)), the last two cosine terms in equation (5.6) cancel and the displacement response reduces to *u*(*t*) = (*F*_0_/*k*_1_)[*U*(*t* − 0) − *U*(*t* − *T*_*e*_)]. On the other hand, when the duration of the rectangular pulse is not an integer multiple of a day (say *T*_*e*_ = 138 hours), the displacement response of a city, *u*(*t*), upon being subjected to the rectangular pulse exhibits perpetual oscillations (figure 14*c*). Consider now a city operating under normal conditions, therefore its MSD 〈*r*^2^(*t*)〉 of its citizens is expressed with equation (4.8). For the simplest admissible mechanical analogue shown in figure 9, the corresponding normalized MSD *k*_1_〈*r*^2^ (*t*)〉/*W* = 〈*r*^2^ (*t*)〉/*A*_*o*_ given by equation (4.8) is plotted at the top of figure 14*a*. While the city operates under normal conditions, it is suddenly subjected to an acute shock at some time *t*_0_ = 0 that is described with the rectangular pulse given by equation (5.1) with a duration *T*_*e*_ as shown in figure 14*b*. In the response analysis shown in figure 14, we have selected a duration of the pulse excitation to be a non-integer multiple of a day (*T*_*e*_ = 138 h = 5 days + 18 h), so that *u*(*t*) as expressed by equation (5.6) exhibits oscillations. The normalized response of the city, *k*_1_*u*(*t*)/*F*_0_, to this rectangular pulse alone is offered by equation (5.6) and plotted in figure 14*c*. Given that in this work, we consider the city to be a linear network, the overall response of the city subjected to the acute shock (rectangular pulse) is the superposition of its steady-state response under normal conditions given by equations (4.7) or (4.8) and shown in figure 14*a* (response to a unit step loading at the distant past that produces the creep compliance, *J*(*t*)) and its response due to the rectangular pulse at time *t*_0_ given by equation (5.6) and shown in figure 14*c*. Accordingly, the ‘post-event’ response of a city as is described with the three-parameter inertoelastic solid is

5.7 $$k_{1}J(t)-\frac{k_{1}}{F_{0}}u(t)=U(t-T_{e})-\frac{\beta }{1+\beta }[\omega_{R}(t-T_{e})]$$

The minus sign (rather than a plus sign) in the left-hand side of equation (5.7) is because the acute shock is expected to suppress the normal activity of the city as expressed by equations (4.7) or (4.8). The right-hand side of equation (5.7) that results after subtracting equation (5.6) from equation (4.7) is precisely of the same form as equation (4.7) and uncovers the remarkable result that upon the expiration of the acute shock (rectangular pulse), the mechanical model shown in figure 9 and expressed by equation (4.3) predicts that the city reverts immediately to its normal activity (same 〈*r*^2^ (*t*)〉 as before the acute shock), implying that cities when subjected to a rectangular pulse of any duration, *T*_*e*_ are inherently and invariably resilient. Figure 14*d* plots the pre- and post-event response as predicted by equation (5.7) and compares the analytical prediction with the recorded response of the Dallas metroplex following the 2021 North America cold storm during the third week of February 2021 for which we have used a pulse excitation duration *T*_*e*_ = 138 h.

**Figure 15.**
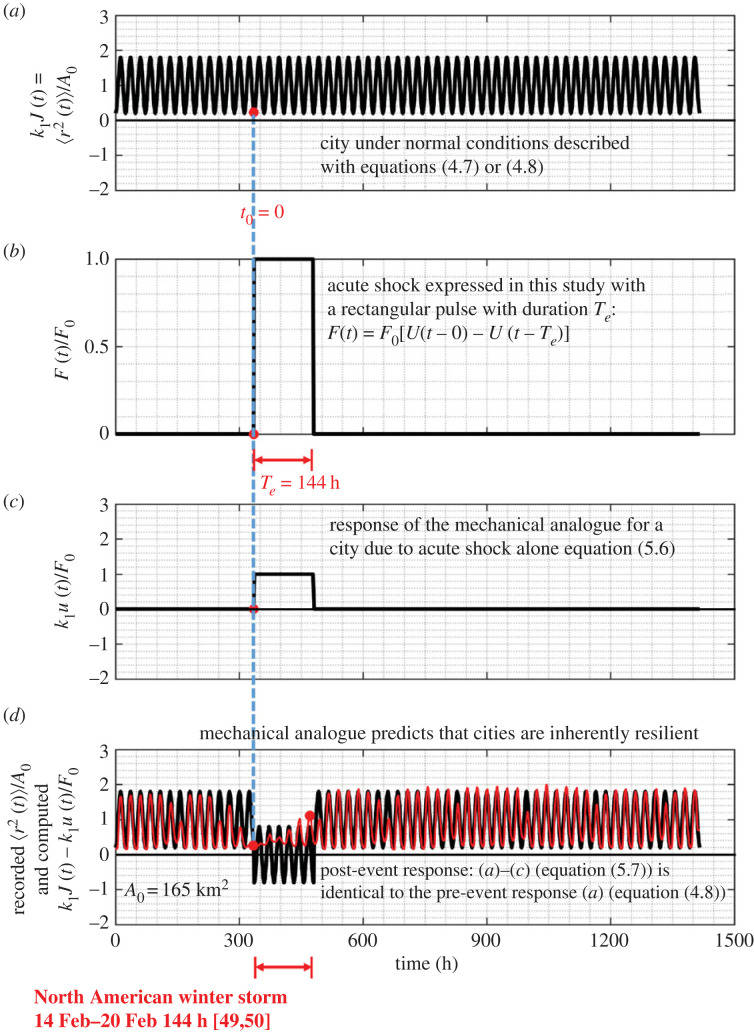
(*a*) Normalized behaviour of a city under normal conditions as given by equations (4.7) or (4.8). (*b*) Acute shock on a city expressed with a rectangular pulse with duration *T*_*e*_ = 144 h that is an integer multiple of a day = 24 h. (*c*) Normalized response of a city to a rectangular pulse alone with duration *T*_*e*_ = 144 h. (*d*) Pre- and post-event response of a city ((*a*–*c*), solid dark lines) which is identical to the pre-event response of the city (*a*). The thin red lines is the normalized MSD of the Dallas metroplex prior, during and after the North American winter storm during the third week of February 2021 as computed from the recorded GPS location data.

The result that cities of average population density such as the Dallas metroplex examined in this study are inherently resilient to acute shocks as indicated by equation (5.7) is also illustrated in figure 15 for the case where the duration of the rectangular shock is an integer multiple of a day (*T*_*e*_ = 144 h) as shown in figure 15*b*.

Given the linearity of the candidate constitutive model for a city expressed by equation (4.3) and illustrated in figure 9, an alternative way to reach equation (5.6) is through the convolution integral

5.8 $$u(t)=\int_{0^{-}}^{t}h(t-\xi )F(\xi )\;d\xi ,$$

where *F*(*ξ*) is the time history of the acute shock—in this analysis the rectangular pulse given by equation (5.1)—and *h*(*t*) is the impulse response function [67–71] that is defined as the resulting displacement history *u*(*t*), due to an impulsive force input *F*(*t*) = *δ*(*t* − *ξ*) with *ξ* < *t*. Given that *δ*(*t* − *ξ*) = d*U*(*t* − *ξ*)/d*t*, the impulse response function of a linear system *h*(*t*) = d*J*(*t*)/d*t* where *J*(*t*) is its creep compliance (displacement history due to a unit step force). Accordingly, the impulse response function of the three-parameter inertoelastic solid described with equation (4.3) and illustrated by figure 7 is

5.9 $$h(t)=\frac{dJ(t)}{dt}=\frac{1}{k_{1}}\left[\delta (t-0)+\frac{\beta }{1+\beta }\omega_{R}\sin(\omega_{R}t)\right],$$

where *J*(*t*) is given by equation (4.7) and *δ*(*t* − 0) is the Dirac delta function [62]. Substitution of the expression of the impulse response function given by equation (5.9) into equation (5.8) after using the forcing function *F*(*ξ*) given by equation (5.1) yields

5.10 $$\begin{array}{rl} u(t) & =\frac{F_{0}}{k_{1}}\left[\int_{0^{-}}^{t}\delta (t-\xi )[U(\xi -0)-U(\xi -T_{e})]\;d\xi +\frac{\beta }{1+\beta }\omega_{R}\int_{0}^{T_{e}}\sin[\omega_{R}(t-\xi )]\;d\xi \right]. \end{array}$$

Using the symmetry of the Dirac delta function *δ*(*t* − *ξ*) = *δ*(*ξ* − *t*) [62], the first integral appearing within the brackets of equation (5.10) gives

5.11 $$\int_{0^{-}}^{t}\delta (\xi -t)[U(\xi -0)-U(\xi -T_{e})]\;d\xi =U(t-0)-U(t-T_{e}),$$

and after expanding the sine function in the second integral, equation (5.10) assumes the form

5.12 $$\begin{array}{rl} u(t) & =\frac{F_{0}}{k_{1}}[U(t-0)-U(t-T_{e})+\frac{\beta }{1+\beta }\omega_{R}[\sin(\omega_{R}t)\int_{0}^{T_{e}}\cos(\omega_{R}\xi )\;d\xi -\cos(\omega_{R}t)\int_{0}^{T_{e}}\sin(\omega_{R}\xi )\;d\xi ]]. \end{array}$$

Upon evaluating the cosine and sine integrals in the right-hand side of equation (5.12), the displacement response history offered by equation (5.6) is recovered.

This second approach, where the displacement response is evaluated with the convolution integral given by equation (5.8), is employed to calculate the response from the amplitude-modulation model for which we do not have a complex frequency response function, H(s) at this time.

### 5.2. Response of the amplitude-modulation model to a rectangular pulse

For a city that the MSD of its citizens 〈*r*^2^ (*t*)〉 is best described with the amplitude-modulation model expressed with equation (4.11) the corresponding creep compliance is

5.13 $$J(t)=\frac{\left\langle r^{2}(t)\right\rangle }{A_{0}k}=\frac{1}{k}[U(t-0)-C[\cos(\omega_{R}t)+\frac{\gamma }{2}\left(\sin\left(\frac{8}{7}\omega_{R}t\right)-\sin\left(\frac{6}{7}\omega_{R}t\right)\right)]]$$

and its impulse response function is

5.14 $$\begin{array}{rl} h(t) & =\frac{dJ(t)}{dt}=\frac{1}{k_{1}}\left[\delta (t-0)+C\omega_{R}\left[\sin(\omega_{R}t)-\frac{\gamma }{2}\left(\frac{8}{7}\cos\left(\frac{8}{7}\omega_{R}t\right)-\frac{6}{7}\cos\left(\frac{6}{7}\omega_{R}t\right)\right)\right]\right]. \end{array}$$

Substitution of the expression of the impulse response function given by equation (5.14) into equation (5.8) after using that the forcing function *F*(*ξ*) is given by equation (5.1), the normalized displacement response from the amplitude-modulation model when subjected to a rectangular pulse of duration *T*_*e*_ is

5.15 $$\begin{array}{rl} \frac{k}{F_{0}}u(t) & =U(t-0)-U(t-t_{e})+C[-\cos(\omega_{R}t)+\cos(\omega_{R}(t-T_{e}))\\ & \;-\frac{\gamma }{2}(\sin\left(\frac{8}{7}\omega_{R}t\right)-\sin\left(\frac{8}{7}\omega_{R}(t-T_{e})\right)-\sin\left(\frac{6}{7}\omega_{R}t\right)+\sin\left(\frac{6}{7}\omega_{R}(t-T_{e})\right))]. \end{array}$$

**Figure 16.**
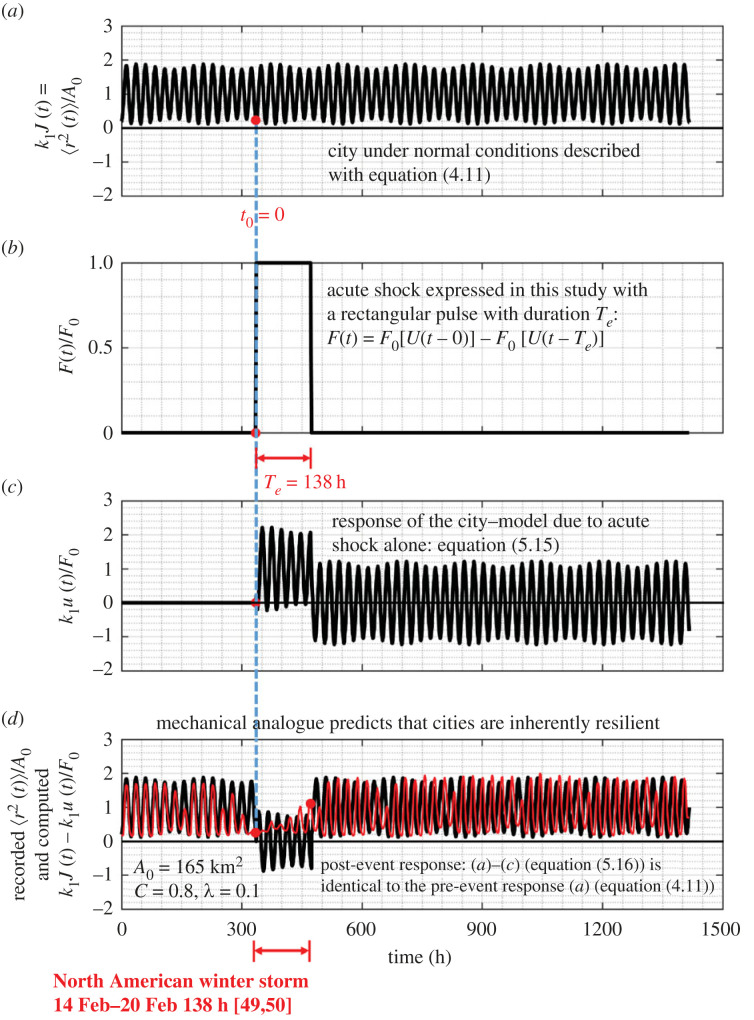
(*a*) Normalized behaviour of a city under normal conditions as given by equations (4.11). (*b*) Acute shock on a city expressed with a rectangular pulse with duration *T*_*e*_ = 138 h that is not an integer multiple of a day = 24 h. (*c*) Normalized response of a city to a rectangular pulse alone with duration *T*_*e*_ = 138 h. (*d*) Pre- and post-event response of a city ((*a*–*c*), solid dark lines) which is identical to the pre-event response of the city (*a*). The thin red lines is the normalized MSD of the Dallas metroplex prior, during and after the North American winter storm during the third week of February 2021 as computed from the recorded GPS location data.

Again, when the duration of the rectangular pulse is not an integer multiple of a day (say *T*_*e*_ = 138 h), the displacement response of a city *u*(*t*) described with the amplitude modulation model exhibits perpetual oscillations as shown in figure 16*c*.

Consider now a city operating under normal conditions and the MSD 〈*r*^2^ (*t*)〉 of its citizens is best described by the amplitude-modulation model given by equation (4.11) and plotted at the top of figure 16*a*. While the city operates under normal conditions, at some time *t*_0_ = 0 is suddenly subjected to an acute shock that is described with a rectangular pulse with duration *T*_*e*_ as shown in figure 16*b*. In the response analysis shown in figure 16, we have selected a duration of the pulse-excitation to be a non-integer multiple of a day (*T*_*e*_ = 138 h = 5 days + 18 h) so that the city response *u*(*t*) as expressed by equation (5.15) exhibits oscillations. The overall response upon the city is subjected to the acute shock (post-event response) is the superposition of its steady-state response under normal conditions given by equation (4.11) and shown in figure 16*a* and its response due to the rectangular pulse given by equation (5.15) and shown in figure 16*c*. Accordingly, the ‘post-event’ response of a city described with the amplitude-modulation model is

5.16 $$\begin{array}{l} kJ(t)-\frac{k}{F_{0}}u(t)=U(t-T_{e})\\ \;\;\;-C\left[\cos[\omega_{R}(t-T_{e})]+\frac{\gamma }{2}\left(\sin\left(\frac{8}{7}\omega_{R}(t-T_{e})\right)-\sin\left(\frac{6}{7}\omega_{R}(t-T_{e})\right)\right)\right]. \end{array}$$

The right-hand side of equation (5.16), that results after subtracting equation (5.15) from equation (4.11), is precisely of the same form as equation (4.11) and confirms our previous finding that, regardless of the sophistication of the linear mathematical model used to approximate the MSD of the citizens of a city, upon the expiration of the acute shock (rectangular pulse), the city reverts immediately to its normal pre-event activities (same 〈*r*^2^ (*t*)〉 as before the acute shock) implying that cities when subjected to a rectangular pulse are inherently resilient within the context of engineering resilience [38].

**Figure 17.**
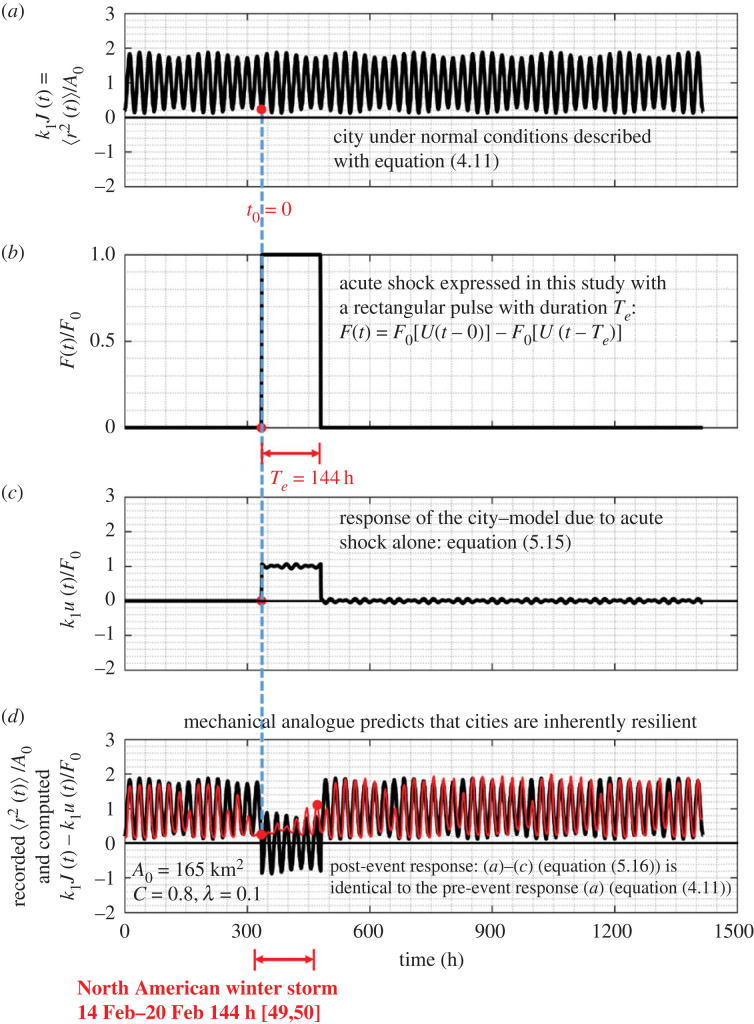
(*a*) Normalized behaviour of a city under normal conditions as given by equation (4.11). (*b*) Acute shock on a city expressed with a rectangular pulse with duration *T*_*e*_ = 144 h that is an integer multiple of a day = 24 h. (*c*) Normalized response of a city to a rectangular pulse alone with duration *T*_*e*_ = 144 h. (*d*) Pre- and post-event response of a city ((*a*–*c*), solid dark lines) which is identical to the pre-event response of the city (*a*). The thin red lines is the normalized MSD of the Dallas metroplex prior, during and after the North American winter storm during the third week of February 2021 as computed from the recorded GPS location data.

The remarkable result—that cities of average population density such as the Dallas metroplex study are inherently resilient to acute shocks, regardless the sophistication of the linear solid-like model that approximates the recorded MSD—is also illustrated in figure 17 for the case where the duration of the rectangular pulse is an integer multiple of a day as shown in figure 17*b*. For the amplitude-modulation model, even when the duration of the rectangular shock is an integer number of days (*T*_*e*_ = 144 h = 6 days) the sine functions appearing in equation (5.15) do not cancel because of the multiplication factors 8/7 and 6/7, resulting to the small undulations that override the rectangular pulse shown in figure 17*c*. The post-event predictions of our mathematical models are in remarkably good agreement with the recorded response of the Dallas metroplex following the 2021 North American winter storm during which over 4 million people lost power due to a resulting state-wide power crisis in Texas causing unforeseen damage to its power grid [54,55].

### 5.3. Accurate estimation of the recovery time of cities by synchronizing the predicted and recorded post-event responses

While our mechanical model predicts with remarkable accuracy the recorded post-event response, it is not capable to capture the local, transient response of a city during the duration of the natural hazard. Our main motivation for developing a mechanical model for cities is for estimating the recovery time of the city when struck by a natural hazard—that is how much time the city needs to revert to its initial steady-state behaviour and resume its normal pre-event activities. Our response analysis framework offers an accurate method for estimating the recovery time of cities when struck by natural hazards by merely taking the duration of the acute pulse, *T*_*e*_, long enough so that the predicted post-event response synchronizes with the recorded post-event response as shown in figure 17*d* (or figure 15*d*). More specifically, figure 16*d* shows that when we used as duration of the rectangular pulse, *T*_*e*_ = 138 h, the predicted MSD of the Dallas metroplex following the storm is identical to the pre-event MSD; yet the predicted post-event MSD is slightly out of phase (6 hours) with the recorded MSD. By contrast, figure 17*d* shows the predicted post-event response of the Dallas metroplex when the duration of excitation of the rectangular pulse is *T*_*e*_ = 6 days = 144 h, synchronizes with the recorded post-event MSD, indicating that the Dallas metroplex recovered essentially immediately from the February 2021 North American winter storm. In the general case, the recovery time, *T*_rec_, is the difference between *T*_*e*_ (duration of the rectangular pulse) and the actual duration of the natural hazard, *T*_nh_, as documented by the federal and state agencies—that is *T*_rec_ = *T*_*e*_ − *T*_nh_. As an example, for the Dallas metroplex, when struck by the February 2021 North American winter storm, figure 17*d* or figure 15*d* suggest that the recovery time is essentially zero (*T*_rec_ = *T*_*e*_ − *T*_nh_ = 0).

## 6. Conclusion

Following the ever-growing number of qualitative studies on urban resilience [13] in association with current trends for developing a quantitative, science-based, predictive framework for the behaviour/response analysis of cities [4,8,25]; in this work, we employ concepts from statistical mechanics and microrheology to develop a mechanical analogue for cities. Our study concentrates on a single-state equilibrium as defined by [38] which is the capacity of a system to revert to its post-disturbance equilibrium state—also known as engineering resilience—and hinges upon the premise that a dependable indicator of the engineering resilience of a city is whether the average mobility pattern of its citizens following an acute shock matches the average mobility pattern before the shock.

We built on the remarkable success of microrheology [27–37] in which the macroscopic frequency and time-response functions of complex viscoelastic materials are extracted by monitoring the thermally driven Brownian motion of probe microparticles suspended within the viscoelastic material, and we computed the MSD (ensemble averages) of thousands of cell-phone users from GPS location data to establish the creep compliance and the resulting impulse response function of a city. The derivation of these time-response functions allows for the synthesis of simple solid-like mechanical analogues with increasing sophistication which are shown to have engineering significance.

Arguments from physical modelling led to the synthesis of the simplest mechanical analogue for a city, which is the three-parameter inertoelastic solid that is a parallel connection of an elastic spring with an inertoelastic fluid (a spring-inerter in-series connection). The three-parameter inertoelastic solid yields a creep compliance which is essentially an elevated cosine that captures the daily oscillations of the MSD of the citizens of a city in association that a city ‘never sleeps’.

Recorded GPS location data from the Dallas metroplex and the San Francisco Bay Area indicate that in addition to the daily oscillations, the MSD of cities exhibits a weekly amplitude-modulation with people moving more during the weekdays and less on Sundays; while, less people are staying at home during the weekends than during the weekdays. These observations motivated the synthesis and response analysis of a solid-like, amplitude-modulation model which captures satisfactorily both the daily and weekly periodicity. Given their linear structure, both the three-parameter inertoelastic model and the more sophisticated amplitude-modulation solid-like model predict that, when a city is subjected to an acute shock that was modelled with a rectangular pulse with arbitrary finite duration, it recovers immediately its initial state (pre-event response), suggesting that cities of average population density, such as the Dallas metroplex examined herein, are inherently resilient within the context of engineering resilience [38]. The mathematical predictions from the mechanical analogues developed in this study confirm similar findings by other investigators, reached with different analysis methodologies, that cities are remarkably robust [8].

## Data Availability

Recorded GPS location datasets for all related graphs reported in this paper are publicly accessible in the Dryad Digital Repository: https://doi.org/doi:10.5061/dryad.1c59zw3zg [72].
